# Urgent need for better quality control, standards and regulation for the Large Language Models used in healthcare domain

**DOI:** 10.3126/nje.v14i2.69361

**Published:** 2024-09-02

**Authors:** Brijesh Sathian, Edwin van Teijlingen, Israel Junior Borges do Nascimento, Russel Kabir, Indrajit Banerjee, Padam Simkhada, Hanadi Al Hamad

**Affiliations:** 1 Geriatrics and long term care department, Rumailah Hospital, Hamad Medical Corporation, Doha, Qatar; 2 Centre for Midwifery& Women’s Health, Bournemouth University, Bournemouth, UK; 3 Division of Country Health Policies and Systems, World Health Organization Regional Office for Europe, Copenhagen, 2100, Denmark; 4 School of Allied Health, Anglia Ruskin University, Essex, UK; 5 Sir Seewoosagur Ramgoolam Medical College, Belle Rive, Mauritius; 6 School of Human and Health Sciences, University of Huddersfield, Huddersfield, UK

## Background

Globally, the increasing elderly population is associated with a rising prevalence of chronic diseases [1]. While advancements in medical care have extended life expectancy, there has not been a commensurate increase in healthy life years. This trend is escalating healthcare costs and compelling governments, insurers, individuals, families, regulators, and healthcare providers to seek innovative solutions and reform healthcare delivery systems worldwide [2]. Furthermore, in the context of the global health crisis, medical systems face the dual challenge of improving performance (delivering effective, high-quality care) while also transforming care delivery on a large scale by incorporating real-world, data-driven insights into patient treatment.

Artificial intelligence (AI) is fundamentally transforming the medical field, with the potential to enhance both physician and patient experiences. Although the transformative impact of AI in healthcare has been anticipated for years, only recently have technological advancements emerged that can capture some of the complexities of health, illness, and healthcare delivery [3, 4]. While large language models (LLMs) have demonstrated remarkable capabilities, the standards for clinical applications remain high. Efforts to evaluate these models' clinical knowledge often rely on automated assessments using narrow criteria. The recent prominence of LLMs in highly visible and interactive applications has generated interest in how emerging AI technologies might benefit medicine and health for patients, the public, physicians, health systems, and other stakeholders [5]. Critical areas of focus should include clinical care and outcomes, patient-centered care, healthcare quality, fairness in AI algorithms, medical education and clinician experience, and global solutions [6].

The potential of artificial intelligence to transform clinical research is substantial, with key advancements required in discovery science and medical applications. Research conducted by Liang et al. has demonstrated that large language models can provide valuable input on research papers [7]. While expert feedback remains essential for precise research, the rapid increase in scholarly output has raised concerns regarding the efficacy of traditional scientific review processes. Obtaining high-quality peer reviews for prestigious journals is becoming increasingly challenging. Studies have revealed a strong correlation between LLM and human feedback in both retrospective and prospective evaluations, as well as positive user perceptions of LLM input's value [7]. Although human expert assessments should continue to be central to scientific processes, Large Language Model (LLM) contributions can support researchers, particularly when timely expert feedback is unavailable or during early manuscript preparation stages. The field of medical artificial intelligence (MAI) has evolved from traditional machine learning to deep learning, progressing towards unsupervised learning models [8]. Recent years have witnessed a shift from task-specific to generalized medical artificial intelligence (GMAI) models. These emerging AI models and algorithms need to be adapted for therapeutic applications across various contexts.

In the past five years, multiple research efforts have demonstrated that MAI models can match or surpass physician performance across various medical tasks and specialties [9-12]. Nevertheless, many of these models have been evaluated only through retrospective analyses using proxy outcomes, without considering real-world clinical environments.

Health information ecosystem data standards play a crucial role in facilitating software integration among healthcare entities for purposes such as data sharing, analysis, clinical research, and public health. However, the potential utilization of large language models to dynamically convert unstructured data into standardized formats for future use raises questions regarding the future of health data [13]. The regulation of clinical artificial intelligence (AI) presents unique challenges for global policymakers [14]. Current methodologies for ensuring AI technology safety and efficacy may be adequate for earlier AI iterations predating generative artificial intelligence (GAI). However, governing clinical GAI may necessitate the development of novel regulatory frameworks. As AI technology advances, researchers, academic institutions, funding bodies, and publishers should continue to examine its impact on scientific inquiry and revise their understanding, ethical guidelines, and regulations accordingly.
